# Feeding of Bait to Snail *Lymnaea acuminata* and Their Effect on Certain Enzyme in the Nervous Tissue

**DOI:** 10.5402/2012/343047

**Published:** 2012-12-04

**Authors:** Pradeep Kumar, V. K. Singh, D. K. Singh

**Affiliations:** Malacology Laboratory, Department of Zoology, Deen Dayal Upadhyay Gorakhpur University, Gorakhpur 273009, India

## Abstract

Fascioliasis, a snail-borne parasitic zoonosis, has been recognized for a long time because of its major veterinary and human impact. Different Bait formulations were fed to the snail *Lymnaea acuminata* in clear glass aquaria having diameter of 30 cm. Snail attractant containing bait formulations was prepared from different binary combination (1 : 1 ratio) of carbohydrates (glucose, starch 10 mM) and amino acid (methionine, histidine 10 mM) in 100 ml of 2% agar solution + sublethal (20% and 60% of 24 h and 96 h LC_50_) doses of different molluscicides (eugenol, ferulic acid, umbelliferone, and limonene). Snails fed on bait containing sub-lethal concentration of different molluscicides and the snail attractant, causing a significant inhibition in alkaline phosphatase (ALP) and acetylcholinesterase (AChE) activity in the nervous tissue of the vector snail *L. acuminata*. Maximum inhibition in ALP (20% of control) and AChE (49.49% of control) activity was observed in the nervous tissue of the *L. acuminata* exposed to 60% of 96 h LC_50_ of eugenol in the bait pellets containing starch + histidine, starch + methionine, respectively.

## 1. Introduction

Fascioliasis is an important cattle and human disease caused by two major species of *Fasciola hepatica* and *F. gigantica* in different parts of the world [1, 2]. Worldwide, 17 million individuals are infected with *Fasciola,* and more than 90 million people are at risk of fascioliasis [3]. Normally, fascioliasis is reported in livestock animals; now, more occurrence of fascioliasis in human population is noted in different parts of the world [4, 5]. In northern India, *Lymnaea acuminata* is the intermediate host of the *Fasciola* species [1, 6–9]. One way to reduce the incidence of fascioliasis is to delink the life cycle of fluke by destroying the intermediate hosts [6, 10–16]. Bait formulation of different molluscicides would be an effective tool for selective killing of the snail with minimal adverse effect on the nontarget animal and environment. The use of a combination of snail attractant and molluscicides in bait formulation [17] is an effective tool for the snails' control. The aim of the present study is to evaluate the effect of sub-lethal feeding of molluscicides eugenol, ferulic acid, umbelliferone, and limonene [14] in bait formulations containing attractant carbohydrates (glucose, starch) and amino acids (methionine, histidine) attractant on alkaline phosphatase (ALP) and acetylcholinesterase (AChE) activity in the nervous tissue of the snail *L. acuminata.*

## 2. Materials and Methods

### 2.1. Collection of Snails

Adult *L. acuminata* (2.25 ± 0.20 cm in length) were collected locally from lakes and low-lying submerged fields. These snails were acclimatized for 72 hours in dechlorinated tap water at 25 ± 1°C. The pH of the water was 7.2–7.3, and dissolved oxygen, free carbon dioxide, and bicarbonate alkalinity were 6.5–7.2 mg/L, 5.2–6.3 mg/L, and 102.0–105.0 mg/L, respectively.

### 2.2. Pure Compounds

Agar-agar, carbohydrates (glucose, starch), amino acids (methionine, histidine), and different active molluscicides such as eugenol, ferulic acid, umbelliferone, and limonene were used in bait formulations. The pure active components, eugenol (2-Methoxy-4-(2-propenyl) phenol), ferulic acid (4-Hydroxy-3-methoxycinnamic), umbelliferone (7-Hydroxy coumarin; 7-hydroxy-2H-1-benzopyran-2-one), and limonene ((R)-4-Isopropenyl-1-methyl-1-cyclohexene), were purchased from Sigma chemical Co., USA. 

### 2.3. Preparation of Bait Formulations

Bait formulations containing binary combination (1 : 1 ratio) of different carbohydrates (glucose, starch 10 mM), amino acids (methionine, histidine 10 mM), and sublethal (20% and 60% of 24 h and 96 h LC_50_) concentration of molluscicides (eugenol, ferulic acid, umbelliferone, and limonene) were prepared in 100 mL of 2% agar solution by the method of Madsen, [18]. Concentrations of carbohydrates and amino acids were based on the earlier reports of Tiwari and Singh [19, 20]. These solutions were spread at a uniform thickness of 5 mm. After cooling, the bait containing sub-lethal molluscicides was cut out by a corer measuring 5 mm in diameter. 

### 2.4. Assay Apparatus and Procedure

The bioassay was performed by the method of Tiwari and Singh [19, 20]. The bioassay chamber consists of a clean glass aquarium having a diameter of 30 cm. Each aquarium was divided into four concentric zones with diameters of 13, 18, 24, and 30 cm: central zone (zone 3), middle zone (zone 2 and 1), and outer zone (zone 0). A small annular elevation of 9 mm height and 2.4 cm in diameter was made in the centre of aquarium (zone 3). Zone 0 had an area of 254 cm^2^ on the periphery of the aquarium. The aquaria were then filled with 500 mL of dechlorinated tap water to a height of 8 mm and maintained at 25 ± 1°C. At the start of the assay, ten individually marked snails of uniform size were placed on the circumference of zone 0. The distance between two snails was 66 mm. Simultaneously, one of the prepared baits containing sublethal concentration of different active molluscicidal components was added on the small annular elevation in the center (zone 3). Six sets of experiments have been designed with ten snails, each for all molluscicides used in this study.

### 2.5. Biochemical Estimations

After 24 h of bait feeding, the snails were washed with water, and the nervous tissue was dissected out from snail brain and used for the measurement of enzyme activities. Alkaline phosphatase (ALP) and acetylcholinesterase (AChE) activity were measured in treated as well as control groups of snails.

In the withdrawal experiment, ALP and AChE activities in the nervous tissue of snail fed on bait formulations were measured in withdrawn snails after 96 h feeding of 60% of 96 h LC_50_ of bait for the next 72 h in fresh water. 

### 2.6. Alkaline Phosphatase Activity

The alkaline phosphatase activity was measured by the method of Bergmeyer, [21] as modified by Singh and Agarwal, [22]. The nervous tissue was homogenized (2% w/v) in ice-cold 0.9% NaCl and centrifuged at 5000 xg for 20 minutes at 4°C. Standard curves were drawn with p-nitrophenol. 0.1 mL of enzyme source supernatant was added in 1.0 mL of alkaline buffer substrate solution (prepared by dissolving 375 mg glycine, 10 mg MgCl_2_·6H_2_O and 165 mg p-nitrophenyl phosphate sodium salt in 42 mL of 0.1 N NaOH, and, mixture was made up to 100 mL with double distilled water). The mixture was mixed thoroughly and incubated for 30 min. at 37°C. 10 mL of 0.02 N NaOH was added to the incubation mixture. The reaction was stopped by the addition of an excess of NaOH. The alkaline phosphatase activity was measured colorimetrically at 420 nm which is a measure of the yellow colour of nitrophenol produced by the hydrolysis of p-nitrophenyl phosphate buffer. The enzyme activity was expressed in moles substrate hydrolyzed/30 min/mg protein.

### 2.7. Acetylcholinesterase

Acetylcholinesterase activity was measured by the method of Ellman et al., [23] as modified by Singh and Agarwal [24]. The nervous tissue of *L. acuminata* was homogenized (50** **mg/mL) in 0.1** **M phosphate buffer (pH 8.0) for 5 minutes in an ice bath and centrifuged at 1000 xg for 30 minutes at 4°C. The clear supernatant was taken as an enzyme source. The enzyme activity was measured in a 10** **mm path-length cuvette using an incubation mixture consisting of 0.1 mL of enzyme source, 2.9 mL of 0.1** **M phosphate buffer (pH 8.0), 0.1 mL of chromogenic agent DTNB (5,5′-dithiobis-(2-nitrobenzoate)) and 0.2 mL of freshly prepared acetylthiocholine iodide. The change in optical density at 412** **nm was continuously observed on spectrophotometer for 3 minutes at 25°C. Enzyme activity was expressed as moles SH hydrolyzed/minute/mg protein.

### 2.8. Statistical Analysis

Each experiment was six times replicated estimation (measurement in six different pool of nervous tissue). The values were expressed as mean ± SE. Student's *t*-test was applied to determine the significant (*P* < 0.05) difference between treated and control animals [25]. 

## 3. Results and Discussion 

Sub-lethal feeding to 20% and 60% of 24 h and 96 h LC_50_ of eugenol, ferulic acid, umbelliferone, and limonene in bait formulations caused a significant (*P* < 0.05) inhibition in alkaline phosphatase activity in the nervous tissue of snail *L. acuminata* (Table 1). Maximum inhibition (20% of control) in alkaline phosphatase activity was observed in the nervous tissue of *L. acuminata* fed on 60% of 96 h LC_50_ of eugenol (Table 1). Significant (*P* < 0.05) recovery in alkaline phosphatase activity was observed in the nervous tissue of* L. acuminata* earlier fed on 60% of 96 h LC_50_ of eugenol bait (20% of control),when discontinued for the next 72 h (34.66% of control). The sub-lethal feeding on 20% and 60% of 24 h and 96 h LC_50_ of eugenol, ferulic acid, umbelliferone, and limonene caused a significant inhibition in the AChE activity in the nervous tissue of the snail *L. acuminata* (Table 2). Maximum inhibition (49.49% of control) in the AChE activity was observed in the nervous tissue of the snail fed on 60% of 96 h LC_50_ of eugenol containing bait (Table 2). There was a significant (*P* < 0.05) recovery in the AChE activity in the nervous tissue of the 72 h withdrawn (56.70%) snails with respect to snails fed on 60% of 96 h LC_50_ of eugenol bait.

The result of the present study indicates that the sub-lethal feeding on 20% and 60% of 24 h and 96 h LC_50_ of the active components, eugenol, ferulic acid, umbelliferone, and limonene, with attractant carbohydrates and amino acids was more effective in killing the *L. acuminata*. Earlier, it has been reported that direct release of eugenol, ferulic acid, umbelliferone, and limonene in aquarium water has significant molluscicidal activity against *L. acuminata* [6, 12]. Kumar et al. [15] have demonstrated that when these active molluscicidal components in bait formulations were fed to snails, they also act as potent molluscicides. Kumar et al. [26] have reported that the combination (1 : 1) of amino acids such as valine + aspartic acid, lysine + valine, lysine + alanine, and alanine + valine with active molluscicides, eugenol, ferulic acid, umbelliferone, and limonene, in bait formulations caused maximum inhibition in ALP (23.57% of control) and AChE (49.48% of control) in nervous tissue of *L. acuminata* exposed to 60% of 96 h LC_50_ of ferulic acid and umbelliferone, respectively. In the present study, the mode of entry of molluscicides into the snail body is through the digestive system. In an earlier study, it was through the body surface, when molluscicides were released directly in water. Although the entry of molluscicide inside the body is different, both methods are equally effective in killing the snails. Snails fed with a sub-lethal dose, that is, 20% and 60% of 24 h and 96 h LC_50_ of different molluscicides, caused a significant inhibition in ALP and AChE activity in the nervous tissue of snail* L. acuminata.* The inhibition in ALP and AChE activities may be due to the direct interference of these active molluscicidal with enzyme. Kumar et al., [27] reported that there was a depletion of amino acid and reduction of protein and nucleic acid level in the ovotestis of *L. acuminata* when these active molluscicides were fed to snails in bait formulations. Alkaline phosphatase plays a critical role in protein synthesis [28], shell formation [29], and other secretary activities [30] and its inhibition may result in the reduction of protein level [22, 31] in gastropods. It plays an important role in the transport of metabolites across the membrane [32]. The AChE inhibition results in the accumulation of acetylcholine at the nerves synapses, so that the post synaptic membrane is in a state of permanent stimulation producing paralysis, ataxia, general lack of coordination in neuromuscular system, and eventual death [26, 33–38]. Animal behavior is a neurotropically regulated phenomenon which is mediated by neurotransmitter substances such as ACh [39]. The enzyme AChE is found in the synaptic regions and mediates the transmission of impulses by breaking acetylcholine into acetic acid and choline [40]. The acetylcholine at neural and neuromotor regions upon accumulation causes hyperexcitability [41] which in turn might also influence behavior pattern of animals. The present study shows that eugenol, ferulic acids, umbelliferone, and limonene that are incorporated in the bait caused significant time- and dose-dependent inhibition in the activities of enzyme, ALP and AChE, in the snails* L. acuminata*. Nagababu et al. [42] reported that eugenol significantly inhibited the rise in SGOT activity and cell necrosis without protecting the endoplasmic reticulum damage as assessed by its failure to prevent a decrease in cytochrome p450 and G-6-phosphatase activities. The inhibitory mechanism implies that eugenol does not inactivate the enzyme directly but may interfere with fatty acid radical intermediate due to its hydroxyl radical scavenging ability and thus plays a role in inhibiting the propagation of lipid peroxidation [43]. Eugenol pretreatment prevents DNA strand break and improves the antioxidant status in thioacetamide-treated rates [44]. Although earlier it has been reported that ferulic acid, umbelliferone, eugenol, and limonene inhibited the ALP and AChE activities in the nervous tissue of snails *L. acuminata* when used directly in aquarium water [12], yet it has been observed in the present study that inhibition of ALP activity in ferulic acid/limonene/eugenol bait fed snails were 1.09/1.26/1.60 times higher than earlier reports of Kumar et al. [12]. This concept is a new technique and approach for the effective control of harmful snails, without using more active molluscicide directly in the water and attracting specifically the particular target snail. 

## Figures and Tables

**Table 1:** Effect of sublethal exposure (20% and 60% of 24 h and 96 h LC_50_) of bait formulations containing eugenol, ferulic acid, umbelliferone, and limonene on the activity of alkaline phosphatase (ALP) in the nervous tissue of the snail *L. acuminata*.

Treatment	24 h LC_50_		96 h LC_50_		Withdrawal
	20%	60%	20%	60%	60% (96 h LC_50_)
Control (Agar)	2.75 ± 0.13 (100)				2.25 ± 0.61 (100)
Control (a) Glu. + Meth.	2.82 ± 0.51 (100)				2.75 ± 0.32 (100)
Control (b) Star. + Meth.	2.65 ± 0.32 (100)				2.61 ± 0.16 (100)
Control (c) Glu. + Hist.	2.75 ± 0.92 (100)				2.64 ± 0.15 (100)
Control (d) Star. + Hist.	2.65 ± 0.82 (100)				2.31 ± 0.25 (100)
Glu. + Meth. + Eug.	1.95 ± 0.06∗ (70.90)	1.32 ± 0.12∗ (48.00)	1.75 ± 0.23∗ (63.63)	1.28 ± 0.72∗ (46.54)	1.99 ± 0.72^+^ (88.44)
Glu. + Meth. + Fer.	1.66 ± 0.25∗ (60.36)	1.25 ± 0.07∗ (45.45)	1.85 ± 0.36∗ (67.27)	1.33 ± 0.20∗ (48.36)	1.75 ± 0.26^+^ (77.77)
Glu. + Meth. + Umb.	1.75 ± 0.11∗ (63.63)	1.55 ± 0.20∗ (56.36)	1.75 ± 0.13∗ (63.63)	1.55 ± 0.09∗ (56.36)	1.86 ± 0.23^+^ (82.66)
Glu. + Meth. + lim.	1.25 ± 0.03∗ (45.45)	1.03 ± 0.26∗ (37.45)	1.85 ± 0.77∗ (67.27)	1.66 ± 0.21∗ (60.36)	1.89 ± 0.73^+^ (84.00)
Star. + Meth. + Eug.	1.98 ± 0.72∗ (72.00)	1.70 ± 0.08∗ (61.81)	1.65 ± 0.26∗ (60.00)	1.60 ± 0.13∗ (58.18)	1.82 ± 0.23^+^ (80.88)
Star. + Meth. + Fer.	1.34 ± 0.06∗ (48.72)	1.22 ± 0.38∗ (44.36)	1.55 ± 0.26∗ (56.36)	1.13 ± 0.07∗ (41.09)	1.38 ± 0.07^+^ (61.33)
Star. + Meth. + Umb.	1.68 ± 0.23∗ (61.09)	1.38 ± 0.16∗ (50.18)	1.40 ± 0.39∗ (50.90)	1.28 ± 0.36∗ (46.54)	1.55 ± 0.38^+^ (68.88)
Star. + Meth. + Lim.	1.35 ± 0.72∗ (49.09)	1.25 ± 0.07∗ (45.45)	1.48 ± 0.21∗ (53.81)	1.28 ± 0.77∗ (46.54)	1.49 ± 0.24^+^ (66.22)
Glu. + hist. + Eug.	1.85 ± 0.11∗ (67.27)	1.63 ± 0.07∗ (59.27)	1.53 ± 0.23∗ (55.63)	1.48 ± 0.20∗ (53.81)	1.65 ± 0.23^+^ (73.33)
Glu. + Hist. + Fer.	1.79 ± 0.38∗ (65.09)	1.58 ± 0.28∗ (57.45)	1.51 ± 0.01∗ (54.90)	1.31 ± 0.19∗ (47.63)	1.52 ± 0.23^+^ (67.55)
Glu. + Hist. + Umb.	1.62 ± 0.25∗ (58.90)	1.35 ± 0.09∗ (49.09)	1.68 ± 0.98∗ (61.09)	1.36 ± 0.22∗ (49.45)	1.49 ± 0.26^+^ (66.22)
Glu. + Hist. + Lim.	1.48 ± 0.63∗ (53.81)	1.25 ± 0.71∗ (45.45)	1.60 ± 0.23∗ (58.18)	1.25 ± 0.85∗ (45.81)	1.51 ± 0.73^+^ (67.11)
Star. + Hist. + Eug.	0.98 ± 0.23∗ (35.63)	0.75 ± 0.03∗ (27.27)	0.65 ± 0.75∗ (23.63)	0.55 ± 0.32∗ (20.00)	0.78 ± 0.23^+^ (34.66)
Star. + Hist. + Fer.	1.23 ± 0.07∗ (44.72)	1.11 ± 0.98∗ (40.36)	1.05 ± 0.60∗ (38.18)	0.99 ± 0.23∗ (36.00)	1.20 ± 0.03^+^ (53.33)
Star. + Hist. + Umb.	1.69 ± 0.08∗ (61.45)	1.68 ± 0.23∗ (61.09)	1.51 ± 0.23∗ (54.90)	1.38 ± 0.75∗ (50.18)	1.59 ± 0.23^+^ (70.66)
Star. + Hist. + Lim.	1.42 ± 0.72∗ (51.63)	1.24 ± 0.29∗ (45.09)	1.48 ± 0.05∗ (53.81)	1.28 ± 0.65∗ (46.54)	1.55 ± 0.26^+^ (69.33)

Each value is mean ± SE of six replicates. Values in parentheses are percent change with control taken as 100%.

Concentration (w/v) has been expressed as final concentration in aquarium water. ∗Significant (*P* < 0.05) when *t*-test was applied in between treated and control groups and ^+^in between 60% of 96 h LC_50_ and withdrawal group. Glu.: glucose; Star.: starch; Meth.: methionine; Hist.: histidine; Eug.: eugenol; Feb.: ferulic acid; Umb.: umbelliferone; Lim.: limonene.

**Table 2:** Effect of sublethal exposure (20% and 60% of 24 h and 96 h LC_50_) of bait formulations containing eugenol, ferulic acid, umbelliferone, and limonene on the activity of acetylcholinesterase (AchE) in the nervous tissue of the snail *L. acuminata*.

Treatment	24 h LC _50_		96 h LC_50_		Withdrawal
	20%	60%	20%	60%	60% (96 h LC_50_)
Control (Agar)	0.099 ± 0.002 (100)				0.097 ± 0.007 (100)
Control (a) Glu. + Meth.	0.098 ± 0.003 (100)				0.097 ± 0.005 (100)
Control (b) Star. + Meth.	0.097 ± 0.002 (100)				0.098 ± 0.006 (100)
Control (c) Glu. + Hist.	0.096 ± 0.005 (100)				0.095 ± 0.001 (100)
Control (d) Star. + Hist.	0.098 ± 0.004 (100)				0.097 ± 0.006 (100)
Glu. + Meth. + Eug.	0.075 ± 0.001∗ (75.75)	0.063 ± 0.002∗ (63.63)	0.05 ± 0.001∗ (58.58)	0.051 ± 0.003∗ (51.51)	0.067 ± 0.006^+^ (69.07)
Glu. + Meth. + Fer.	0.065 ± 0.003∗ (65.65)	0.051 ± 0.002∗ (51.51)	0.086 ± 0.003∗ (86.86)	0.065 ± 0.004∗ (65.65)	0.078 ± 0.003^+^ (98.73)
Glu. + Meth. + Umb.	0.073 ± 0.001∗ (73.73)	0.068 ± 0.008∗ (68.68)	0.070 ± 0.003∗ (70.70)	0.055 ± 0.001∗ (55.55)	0.063 ± 0.001^+^ (64.94)
Glu. + Meth. + lim.	0.078 ± 0.002∗ (78.78)	0.069 ± 0.001∗ (69.69)	0.067 ± 0.002∗ (67.67)	0.060 ± 0.003∗ (60.60)	0.075 ± 0.002^+^ (77.31)
Star. + Meth. + Eug.	0.055 ± 0.006∗ (55.55)	0.050 ± 0.003∗ (50.50)	0.058 ± 0.005∗ (58.58)	0.049 ± 0.006∗ (49.49)	0.055 ± 0.004^+^ (56.70)
Star. + Meth. + Fer.	0.062 ± 0.004∗ (62.62)	0.060 ± 0.008∗ (60.60)	0.058 ± 0.001∗ (58.58)	0.054 ± 0.003∗ (54.54)	0.059 ± 0.003^+^ (60.82)
Star. + Meth. + Umb.	0.072 ± 0.005∗ (71.71)	0.065 ± 0.001∗ (65.65)	0.062 ± 0.002∗ (62.62)	0.057 ± 0.005∗ (57.57)	0.062 ± 0.003^+^ (63.91)
Star. + Meth. + Lim.	0.072 ± 0.008∗ (72.72)	0.063 ± 0.004∗ (63.63)	0.068 ± 0.003∗ (68.68)	0.058 ± 0.002∗ (58.58)	0.062 ± 0.001^+^ (63.91)
Glu. + hist. + Eug.	0.057 ± 0.001∗ (57.57)	0.054 ± 0.002∗ (54.54)	0.055 ± 0.007∗ (55.55)	0.050 ± 0.007∗ (50.50)	0.058 ± 0.006^+^ (59.79)
Glu. + Hist. + Fer.	0.082 ± 0.003∗ (82.82)	0.075 ± 0.006∗ (75.75)	0.069 ± 0.003∗ (69.69)	0.061 ± 0.003∗ (61.61)	0.069 ± 0.003^+^ (71.13)
Glu. + Hist. + Umb.	0.070 ± 0.008∗ (70.70)	0.068 ± 0.001∗ (68.68)	0.069 ± 0.004∗ (69.69)	0.066 ± 0.002∗ (66.66)	0.070 ± 0.006^+^ (72.16)
Glu. + Hist. + Lim.	0.069 ± 0.003∗ (69.69)	0.066 ± 0.003∗ (66.66)	0.065 ± 0.007∗ (65.65)	0.060 ± 0.003∗ (60.60)	0.006 ± 0.003^+^ (68.04)
Star. + Hist. + Eug.	0.057 ± 0.006∗ (57.57)	0.055 ± 0.004∗ (55.55)	0.055 ± 0.003∗ (55.55)	0.051 ± 0.005∗ (51.51)	0.057 ± 0.008^+^ (58.76)
Star. + Hist. + Fer.	0.060 ± 0.003∗ (60.60)	0.059 ± 0.003∗ (59.59)	0.058 ± 0.002∗ (58.58)	0.055 ± 0.001∗ (55.55)	0.058 ± 0.002^+^ (59.79)
Star. + Hist. + Umb.	0.078 ± 0.008∗ (78.78)	0.077 ± 0.006∗ (77.77)	0.075 ± 0.001∗ (75.75)	0.070 ± 0.003∗ (70.70)	0.073 ± 0.006^+^ (75.25)
Star. + Hist. + Lim.	0.074 ± 0.003∗ (74.74)	0.071 ± 0.002∗ (71.71)	0.072 ± 0.002∗ (72.72)	0.068 ± 0.004∗ (68.68)	0.073 ± 0.003^+^ (75.25)

Each value is mean ± SE of six replicates. Values in parentheses are percent change with control taken as 100%.

Concentration (w/v) has been expressed as final concentration in aquarium water. ∗Significant (*P* < 0.05) when *t*-test was applied in between treated and control groups and ^+^in between 60% of 96 h LC_50_ and withdrawal group. Glu.: glucose; Star.: starch; Meth.: methionine; Hist.: histidine; Eug.: eugenol; Feb.: ferulic acid; Umb.: umbelliferone; Lim.: limonene.

## References

[1] Agarwal R. A., Singh D. K. (1988). Harmful gastropods and their control. *Acta Hydrochimica et Hydrobiologica*.

[2] Mas-Coma S., Valero M. A., Bargues M. D. (2009). Chapter 2 fasciola, lymnaeids and human fascioliasis, with a global overview on disease transmission, epidemiology, evolutionary genetics, molecular epidemiology and control. *Advances in Parasitology*.

[3] Keiser J., Utzinger J. (2009). Food-borne trematodiases. *Clinical Microbiology Reviews*.

[4] Chen M. G., Mott K. E. (1990). Progress in assessment of morbidity due to *Fasciola hepatica* infection: a review of recent literature. *Tropical Diseases Bulletin*.

[5] Mas-Coma M. S., Esteban J. G., Bargues M. D. (1999). Epidemiology of human fascioliasis: a review and proposed new classification. *Bulletin of the World Health Organization*.

[6] Kumar P., Singh D. K. (2006). Molluscicidal activity of *Ferula asafoetida*, *Syzygium aromaticum* and *Carum carvi* and their active components against the snail *Lymnaea acuminata*. *Chemosphere*.

[7] Sunita K., Singh D. K. (2011). Fascioliasis control: in vivo and in vitro phytotherapy of vector snail to kill *Fasciola* larva. *Journal of Parasitology Research*.

[8] Singh K. K., Singh D. K., Singh V. K. (2012). Toxicity of *Bauhinia variegata* and *Mimusops elengi* with plant molluscicides against *Lymnaea acuminata*. *Journal of Biology and Earth Science*.

[9] Sunita K., Kumar P., Singh D. K. (2012). Abiotic environmental factors and infection of *Fasciola gigantica* in vector snail *Lymnaea acuminata*. *Researcher*.

[10] Godan D. (1983). *Pests Slugs and Snail Biology and Control, Edited By Dora Godan, Translated By Sheila Gruber*.

[11] Marston A., Hostettmann K. (1985). Review article number 6. Plant molluscicides. *Phytochemistry*.

[12] Kumar P., Singh V. K., Singh D. K. (2009). Kinetics of enzyme inhibition by active molluscicidal agents ferulic acid, umbelliferone, eugenol and limonene in the nervous tissue of snail *Lymnaea acuminata*. *Phytotherapy Research*.

[13] Kumar P., Singh D. K. (2010). Amino acids and carbohydrates binary combination as an attractant in bait formulation against the snail *Lymnaea acuminata*. *Malaysian Applied Biology*.

[14] Kumar P., Singh V. K., Singh D. K. (2011). Combination of molluscicides with attractant carbohydrates and amino acids in bait formulation against the snail *Lymnaea acuminata*.. *European Review for Medical and Pharmacological Sciences*.

[15] Kumar P., Singh V. K., Singh D. K. (2011). Bait formulation of molluscicides with attractant amino acid against the snail Indoplanorbis exustus. *Pharmacologyonline*.

[16] Kumar P., Singh V. K., Singh D. K. (2012). Attractant food pellets containing molluscicides against the fresh water snail Indoplanorbis exustus. *Global Veterinaria*.

[17] Kumar P., Singh D. K. (2007). Binary combination of some common species against harmful snail. *Journal of Applied Bioscience*.

[18] Madsen H. (1992). A comparative study on the food-locating ability of *Helisoma duryi*, *Biomphalaria camerunensis* and *Bulinus truncatus* (Pulmonata: Planorbidae). *Journal of Applied Ecology*.

[19] Tiwari F., Singh D. K. (2004). Attraction to amino acids by *Lymnaea acuminata*, the snail host of *Fasciola* species. *Brazilian Journal of Medical and Biological Research*.

[20] Tiwari F., Singh D. K. (2004). Behavioural responses of the snail *Lymnaea acuminata* to carbohydrates in snail-attractant pellets. *Naturwissenschaften*.

[21] Bergmeyer U. H. (1967). *Methods of Enzymatic Analysis*.

[22] Singh D. K., Agarwal R. A. (1989). Toxicity of piperonyl butoxide-carbaryl synergism on the snail *Lymnaea acuminata*. *Internationale Revue der Gesamten Hydrobiologie*.

[23] Ellman G. L., Courtney K. D., Andres V., Featherstone R. M. (1961). A new and rapid colorimetric determination of acetylcholinesterase activity. *Biochemical Pharmacology*.

[24] Singh D. K., Agarwal R. A. (1983). *In vivo* and *in vitro* studies on synergism with anticholinesterase pesticides in the snail *Lymnaea acuminata*. *Archives of Environmental Contamination and Toxicology*.

[25] Sokal R. R., Rohlf F. J. (2007). *Introduction of Biostatistics*.

[26] Kumar P., Singh V. K., Singh D. K. (2012). Enzyme activity in the nervous tissue of *Lymnaea acuminata* fed to different bait formulations. *American Journal of Chemistry*.

[27] Kumar P., Singh V. K., Singh D. K. (2011). Bait formulations of molluscicides and their effects on biochemical changes in the ovotestis of snail *Lymnaea acuminata* (Mollusca, Gastropoda:Lymneidae). *Revista do Instituto de Medicina Tropical de Sao Paulo*.

[28] Pilo B., Asnani M. V., Shah R. V. (1972). Studies on wound healing and repair in pigeon liver. III. Histochemical studies on the acid and alkaline phosphatases during the processes. *Journal of Animal Morphology and Physiology*.

[29] Timmermans L. P. M. (1969). Studies on shell formation in mollusks. *Netherlands Journal of Zoology*.

[30] Ibrahim A. M., Higazi M. G., Demian E. S. (1974). Histochemical localization of alkaline phosphatase activity in the alimentary tract of the snail *Marisa coruarielis*. *Bulletin of the Zoological Society of Egypt*.

[31] Singh K., Singh D. K. (1995). Effect of Azadirachta indica (Neem) on the biochemical parameters in the ovotestis of *Lymnaea acuminata*. *Malaysia Applied Biology*.

[32] Vorbrodt A. (1959). The role of phosphate in intracellular metabolism. *Postepy Higieny i Medycyny Doswiadczalnej*.

[33] Matsumura F. (1985). *Toxicology of Insecticides*.

[34] Singh V. K., Singh S., Singh S., Singh D. K. (1999). Effect of active molluscicidal component of spices on different enzyme activities and biogenic amin levels in the nervous issue of *Lymnaea acuminata*. *Phytotherapy Research*.

[35] Singh S., Singh D. K. (2000). Effect of active molluscicidal components *Abrus precatorius*, *Argemone mexicana* and *Nerium indicum* on certain enzymes in the nervous tissue of *Lymnaea acuminata*. *Journal of Sciences*.

[36] Tripathi S. M., Singh V. K., Singh S., Singh D. K. (2004). Enzyme inhibition by the molluscicidal agent *Punica granatum* Linn. bark and *Canna indica* Linn. root. *Phytotherapy Research*.

[37] Shukla S., Singh V. K., Singh D. K. (2006). The effect of single, binary, and tertiary combination of few plant derived molluscicides alone or in combination with synergist on different enzymes in the nervous tissues of the freshwater snail *Lymnaea (Radix) acuminata* (Lamark). *Pesticide Biochemistry and Physiology*.

[38] Jaiswal P., Singh V. K., Singh D. K. (2008). Enzyme inhibition by molluscicidal component of Areca catechu and Carica papaya in the nervous tissue of vector snail *Lymnaea acuminata*. *Pesticide Biochemistry and Physiology*.

[39] Bullock, Orkand T. H., Grinnell A R. (1977). *Introduction to Nervous Systems*.

[40] O'Brien R. D., Wilkinson C. F. (1976). Acetylcholinesterase and its inhibition. *Insecticide Biochemistry and Physiology*.

[41] Kabeer Ahammad Sahib I., Ramana Rao K. V. (1980). Toxicity of malathion to the freshwater fish *Tilapia mossombica*. *Bulletin of Environmental Contamination and Toxicology*.

[42] Nagababu E., Rifkind J. M., Boindala S., Nakka L. (2010). Assessment of antioxidant activity of eugenol in vitro and *in vivo*. *Methods in Molecular Biology*.

[43] Naidu K. A. (1995). Eugenol-an inhibitor of lipoxygenase-dependent lipid peroxidation. *Prostaglandins Leukotrienes and Essential Fatty Acids*.

[44] Yogalakshmi B., Viswanathan P., Anuradha C. V. (2010). Investigation of antioxidant, anti-inflammatory and DNA-protective properties of eugenol in thioacetamide-induced liver injury in rats. *Toxicology*.

